# Supplementary material for: FoMo: A unifying theory of visual foraging
Source: PLoS Comput Biol. 2026 May 12;22(5):e1014266. doi: 10.1371/journal.pcbi.1014266 (PMC13186390; doi:10.1371/journal.pcbi.1014266)
Supplement: Attachment — Submitted filename: FOMO_REV_BGGL.pdf [file pcbi.1014266.s001.pdf]

I was invited to review the manuscript entitled **"FoMo: A Unifying Theory of Visual Foraging"** for *PLOS Computational Biology*. Overall, I consider this to be a strong and timely contribution that will advance the study and understanding of visual foraging tasks within cognitive psychology and computational science.

The manuscript proposes a unifying theoretical framework that integrates and extends prior approaches to visual foraging. Importantly, it introduces two additional parameters to the original FoMo model to account for spatial aspects that had not been explicitly considered in earlier versions. This extension is particularly relevant given that visual foraging experiments are typically conducted in two-dimensional spatial environments, where the structure and organization of space can meaningfully shape behavior.

The paper also appropriately acknowledges broader theoretical questions regarding when foragers decide to stop searching or leave a patch, a central issue historically associated with Charnov's Marginal Value Theorem (MVT). While the present manuscript does not resolve ongoing debates about the applicability of MVT to human foraging, it recognizes this point as important and aligns well with recent work suggesting that spatial and organizational factors may be critical for understanding stop/leave decisions in humans. In this sense, incorporating spatial parameters into the FoMo framework represents a valuable step that may help guide future work on these questions, even if it does not fully settle them.

The manuscript is very clearly written and reads smoothly throughout. The theoretical review is excellent; well structured, comprehensive, and persuasive; and the motivation and relevance of the topic are presented convincingly. The results are communicated clearly, and the discussion is thoughtful and well aligned with the proposed framework and the broader literature.

Finally, I note a limitation of my own review: I have been able to evaluate the manuscript thoroughly from a theoretical and conceptual standpoint, but I am not an expert in the mathematical details underlying the model parameters, which constrains the depth of my assessment in that respect. I expect that other reviewers with greater technical expertise will be able to provide more detailed evaluation of the formal and mathematical aspects. That said, I will include a few targeted comments aimed at improving clarity for readers who may not have a strong mathematical background.

Overall, I have little to criticize at a general level. I have no doubt that the manuscript is suitable for publication in *PLOS Computational Biology*. My comments are limited to a small number of minor issues, which I detail below; accordingly, my recommendation is **minor revision**, as the core contributions are solid and very well articulated.

Below, I present a number of minor comments and suggestions intended to improve clarity and accessibility. For ease of review, these comments are organized following the structure of the manuscript, from the Introduction through the Discussion. This ordering is purely for convenience and does not reflect the relative importance of the points raised. One caveat is that the first two comments are more general in nature and therefore appear first, before the section-by-section remarks.

1. Notation in mathematical expressions

Throughout the manuscript, the notation used in several formulas can be difficult to follow, particularly for readers who are less familiar with mathematical modeling. While I understand that some definitions may have been omitted to improve readability, in several cases it is not clear what specific notations (e.g., *r*, *i*, *j*, *K*, etc.) refer to. Sometimes these are explained, but at other times they are not, or the explanation appears earlier and is easy to miss.

I believe the manuscript would benefit from a systematic revision of the equations to ensure that **all parameters and symbols are explicitly defined at the point where they are introduced**, for example using formulations such as: *"where K denotes ..., and j represents the number of iterations,"* etc. This would greatly facilitate comprehension, especially for readers with a stronger theoretical than mathematical background, and would make the model more accessible overall.

2. Accessibility and practical use of the model

The manuscript states that the FoMo framework is intended to be clear and usable by the broader community; however, the complexity of the presentation may unintentionally discourage researchers from actually implementing the model. I suggest that the authors make it very explicit where the scripts are located and how they can be used, ideally providing a simplified guide or example workflow (even if placed in an appendix or supplementary materials).

While this point is briefly mentioned in the Conclusions, I feel that offering more concrete guidance would be extremely valuable. Otherwise, there is a risk that the model remains a strong theoretical contribution without being widely adopted. Facilitating implementation seems crucial if the goal is to encourage future use and extension of the model by other researchers.

Introduction

1. **Individual differences, development, and MVT-related findings (p. 4, lines 363–366)**

On page 4, the authors discuss individual differences in visual foraging and note that FoMo can detect such differences, particularly in developmental contexts (lines 363–366). When discussing Marginal Value Theorem–related aspects, however, the Introduction does not note that evidence for developmental differences in MVT-related measures in humans is currently limited (e.g., Gil-Gómez de Liaño et al., 2022; Gil-Gómez de Liaño & Wolfe, 2022).

As a purely optional contextual clarification, the authors could briefly mention that developmental differences are not always observed in MVT-related outcomes. This contrast may be particularly interesting given evidence that spatial organization shows developmental differences (e.g., article 56 cited in the manuscript), and that spatial organization may predict leave/stop decisions in visual foraging better than MVT, as shown in a recent work (Bella et al., 2025).

I emphasize that this is intended as a conceptual suggestion rather than a request to include specific citations. If the authors find it useful, acknowledging this contrast either

in the Introduction (p. 4) or later in the Discussion (p. 17, lines 1151–1158) could further strengthen the theoretical motivation for the spatial parameters introduced in the model.

(References for these studies can be provided below if the authors find this suggestion useful.)

2. **Minor typographical issues in the intro**

- Page 5, line 344: the phrase *"differences in"* appears to contain an extra *"in"* and may need correction.
- Page 7, line 508: the word *"next"* appears to be unnecessary.
- Figure 4 caption (page 7), third line: *"fleixbly"* should be corrected to *"flexibly."*

Methods

1. Justification of the value of K (p. 8, line 555; p. 9)

Around page 8 (line 555), it is stated that K is determined manually, and later (on page 9) it is specified that $K = 20$. However, the rationale for choosing this specific value is not explained. Although the Results section later shows that different values of K do not substantially affect the outcomes under the current model, the initial choice of 20 still appears somewhat arbitrary without further justification. I suggest briefly explaining why this value was selected, either at its first mention (p. 8) or when it is specified numerically (p. 9). Providing this explanation would improve transparency and prevent confusion for readers.

2. Use of the terms "trial" and "patch"

A more general issue that applies to both the Methods and Results sections concerns the use of the terms *trial* and *patch*. My understanding is that these concepts are closely related, and that a *trial* may effectively correspond to what is typically referred to as a *patch* in the foraging literature. However, this equivalence is not explicitly stated.

Clarifying this relationship at some point in the Methods would be helpful. If the terms are not strictly equivalent, a brief explanation of the distinction would also be beneficial. This issue becomes particularly confusing on page 10, where trials are discussed and the phrase *"as opposed to in patches"* (line 683) may lead to ambiguity, at least for readers with a background in foraging theory.

Results

1. Clarification of chance-level performance (p. 11, line 801)

On page 11 (line 801), the text refers to performance being *"around 50%."* While this information is reported in Table 3, I suggest adding a brief clarification such as *"when chance performance is below 0.12 in all cases"* (or similar wording). This additional context would make the result easier to interpret at first glance. The point is explained

clearly later in the Discussion, but including it here would make the Results section more informative and self-contained.

2. Reminding the reader of model differences across the Results

At some points in the Results section, it becomes slightly difficult to keep track of what each model variant contributes. In particular, Model 1.3 (arguably the best-performing model and the one that incorporates absolute direction) could be briefly reintroduced or reminded to the reader at key points throughout the section. This would help maintain clarity when interpreting the comparative results. This is a very minor suggestion, and the authors should feel free to adopt it only if they find it useful.

3. Figure 6 caption

The caption of Figure 6 would benefit from additional detail. Although the main text explains the figure, it would be helpful if the caption itself included more information, particularly regarding what is being measured on the axes in the lower panels. In addition, briefly reminding the reader what parameters such as *b_a*, *b_s*, etc., represent in the four upper panels would improve accessibility and allow the figure to stand more independently from the text.

4. Table 4 caption

A similar comment applies to the caption of Table 4. While some information is provided, adding a bit more detail to explain what each value represents would facilitate interpretation, especially for readers who may consult the table without reading the surrounding text in detail.

5. Clarity of Figures 7 and 9

In Figure 7, it would be helpful to explicitly state in the caption that the white lines represent the search path and that the triangles correspond to targets (this is my interpretation). Without this clarification, the intended message of the figure is difficult to grasp. A similar issue arises in Figure 9. These are minor presentation issues, but clarifying these elements would improve the overall comprehensibility of the results.

Overall, these are minor figure-related suggestions aimed solely at improving clarity and interpretability; addressing them would further strengthen the presentation of the Results section.

Discussion

1. Search strategies as a potential future direction

The Discussion is very well written and thoughtfully connects the findings to the broader literature. One aspect that I found particularly stimulating is the discussion of search strategies. This prompted the thought that it might be valuable in future work to explicitly manipulate or simulate different search strategies (e.g., spiral search, left-to-right scanning resembling reading) and examine how the model responds to these organizational patterns. This could involve instructing participants to adopt specific

strategies or implementing such strategies in simulations. I offer this purely as a conceptual suggestion.

2. Dynamic foraging environments

In the section on Future Directions, it might be worth briefly mentioning dynamic foraging environments. There is a substantial literature on dynamic in-movement foraging, and a short discussion of how the current framework could be extended to situations in which targets or environments change and move around could broaden the scope of the paper without requiring additional analyses. Again, this is intended as a suggestion rather than a requirement.

3. Two-dimensional versus three-dimensional environments

Finally, while the focus on two-dimensional space is entirely appropriate given the experimental paradigms considered, it may be useful to briefly acknowledge that real-world foraging often occurs in three-dimensional environments. Highlighting this point would help situate the current model as an important first step, while recognizing potential extensions to more ecologically realistic settings.

TO CONCLUDE, I reiterate that this is a **very strong manuscript** that makes a valuable and timely contribution. The comments above are minor and primarily intended to improve clarity and accessibility. In my view, the article clearly merits publication and will be of considerable interest to the scientific community.

Refs.

Bella-Fernández, M., Suero Suñé, M., Ferrer-Mendieta, A., Gil-Gómez de Liaño, B. (2025). One factor to bind them all: visual foraging organization to predict patch leaving behavior with ROC curves. *Cognitive Research: Principles & Implications, 10,* 16. https://doi.org/10.1186/s41235-025-00624-7

Gil-Gómez de Liaño, B., Muñoz-García, A., Perez-Hernandez, E., & Wolfe, J. M. (2022). Quitting rules in hybrid foraging search: From early childhood to early adulthood. *Cognitive Development, 64,* 1-14. https://doi.org/10.1016/j.cogdev.2022.101232

Gil-Gómez de Liaño, B. & Wolfe, J. M. (2022). The FORAGEKID Game: Hybrid-Foraging as a new way to study aspects of executive function in development. *Cognitive Development, 64,* 1-14. https://doi.org/10.1016/j.cogdev.2022.101233
