# Supplementary material for: FoMo: A unifying theory of visual foraging
Source: PLoS Comput Biol. 2026 May 12;22(5):e1014266. doi: 10.1371/journal.pcbi.1014266 (PMC13186390; doi:10.1371/journal.pcbi.1014266)
Supplement: Attachment — Submitted filename: response_to_reviewers.pdf [file pcbi.1014266.s003.pdf]

We thank the reviewers for their very helpful comments on the manuscript. Please see our responses below (in red). Page/line numbers refer to the track changes version of the manuscript (where the additions/edits we have made appear in blue).

Reviewer 1

Generally speaking I have been enthusiastic about the approach that this group uses and the modelling approach that they presented recently. They now provide a fine addition to their previous modelling. This is for sure a nice continuation of their work until now, that continues their previous work in a logical way.

So at this point, I do not have many comments, and I think that this is a good contribution to the literature. I also think that their coverage of the literature is quite comprehensive, but I have comments, however (see below)

Here, the authors add a spatial component to their previous model and that is why the authors call this a "unifying" model of foraging. The idea behind the model being "unifying" is that it can explain data from differing paradigms. There is a very important component missing, if this is to be comprehensive, however. Any forager in the real world will face a constantly dynamic, changing scene. Of course, this adds massive computational complexity, but this, nevertheless cannot go unaddressed (see below).

I would encourage the authors to speculate, if not implement how their model would work in a real-world setting. They do rightfully mention that foraging tasks are a pretty good analogue to real-world scenarios. For humans, the main tasks involve gathering information, the frontal lobe equivalent of more basic tectum-based chicken foraging.

While I do not claim that the authors are stuck within their own paradigm, I do encourage them to try to take this to more general scenarios. Let's say I try to cross a busy street. For this, I need to gather lots of information (forage for information), how does the model help us understand how that might occur? The "problem" is that these are non-dynamic scenes. Foraging during continuously changing scenes, must therefore be on the menu? I highlight Thornton et al. (https://link.springer.com/article/10.1186/s41235-021-00299-w; see also https://journals.plos.org/plosone/article?id=10.1371/journal.pone.0219827) as an important consideration – these paradigms get closer to real world scenarios, in my opinion, and that is what the authors in the current manuscript are after. I am not asking the authors to explicitly model theses sort of paradigms, but to speculate what the next steps could look like.

For continuously changing scenes, please see our response to R2 who raises a similar point. We completely agree that moving towards more realistic scenarios should be a

goal of this research, and indeed, we have a recent pre-print led by our PhD student (https://osf.io/preprints/psyarxiv/v3jz4_v1) that is attempting to apply a version of FoMo to a set-up fairly similar to the 'serious game' detailed in Prpic et al. We have now added a paragraph at the end of the discussion (L1211) that considers modelling real-world foraging as a future goal for FoMo.

One minor point but could be important is that the authors refer to summary statistics regarding previous treatment of foraging results. The term "summary statistics" is quite loaded in the literature on visual cognition. So I simply want to warn the authors that many people may think of how ensembles are summarized in perception when they hear this term.

Thank you, this is a very good point: we have reworded to "descriptive statistics" throughout the manuscript.

Reviewer #2:

I was invited to review the manuscript entitled "FoMo: A Unifying Theory of Visual Foraging" for PLOS Computational Biology. Overall, I consider this to be a strong and timely contribution that will advance the study and understanding of visual foraging tasks within cognitive psychology and computational science.

The manuscript proposes a unifying theoretical framework that integrates and extends prior approaches to visual foraging. Importantly, it introduces two additional parameters to the original FoMo model to account for spatial aspects that had not been explicitly considered in earlier versions. This extension is particularly relevant given that visual foraging experiments are typically conducted in two-dimensional spatial environments, where the structure and organization of space can meaningfully shape behavior. The paper also appropriately acknowledges broader theoretical questions regarding when foragers decide to stop searching or leave a patch, a central issue historically associated with Charnov's Marginal Value Theorem (MVT). While the present manuscript does not resolve ongoing debates about the applicability of MVT to human foraging, it recognizes this point as important and aligns well with recent work suggesting that spatial and organizational factors may be critical for understanding stop/leave decisions in humans. In this sense, incorporating spatial parameters into the FoMo framework represents a valuable step that may help guide future work on these questions, even if it does not fully settle them.

The manuscript is very clearly written and reads smoothly throughout. The theoretical review is excellent; well structured, comprehensive, and persuasive; and the motivation

and relevance of the topic are presented convincingly. The results are communicated clearly, and the discussion is thoughtful and well aligned with the proposed framework and the broader literature.

Finally, I note a limitation of my own review: I have been able to evaluate the manuscript thoroughly from a theoretical and conceptual standpoint, but I am not an expert in the mathematical details underlying the model parameters, which constrains the depth of my assessment in that respect. I expect that other reviewers with greater technical expertise will be able to provide more detailed evaluation of the formal and mathematical aspects. That said, I will include a few targeted comments aimed at improving clarity for readers who may not have a strong mathematical background.

Overall, I have little to criticize at a general level. I have no doubt that the manuscript is suitable for publication in PLOS Computational Biology. My comments are limited to a small number of minor issues, which I detail below; accordingly, my recommendation is minor revision, as the core contributions are solid and very well articulated.

Below, I present a number of minor comments and suggestions intended to improve clarity and accessibility. For ease of review, these comments are organized following the structure of the manuscript, from the Introduction through the Discussion. This ordering is purely for convenience and does not reflect the relative importance of the points raised. One caveat is that the first two comments are more general in nature and therefore appear first, before the section-by-section remarks.

1. Notation in mathematical expressions 2

Throughout the manuscript, the notation used in several formulas can be difficult to follow, particularly for readers who are less familiar with mathematical modeling. While I understand that some definitions may have been omitted to improve readability, in several cases it is not clear what specific notations (e.g., r, i, j, K, etc.) refer to. Sometimes these are explained, but at other times they are not, or the explanation appears earlier and is easy to miss. I believe the manuscript would benefit from a systematic revision of the equations to ensure that all parameters and symbols are explicitly defined at the point where they are introduced, for example using formulations such as: “where K denotes ..., and j represents the number of iterations,” etc. This would greatly facilitate comprehension, especially for readers with a stronger theoretical than mathematical background, and would make the model more accessible overall.

We have added a table of notation with definitions (table 1, page 7).

2. Accessibility and practical use of the model

The manuscript states that the FoMo framework is intended to be clear and usable by the broader community; however, the complexity of the presentation may unintentionally discourage researchers from actually implementing the model. I suggest that the authors make it very explicit where the scripts are located and how they can be used, ideally providing a simplified guide or example workflow (even if placed in an appendix or supplementary materials). While this point is briefly mentioned in the Conclusions, I feel that offering more concrete guidance would be extremely valuable. Otherwise, there is a risk that the model remains a strong theoretical contribution without being widely adopted. Facilitating implementation seems crucial if the goal is to encourage future use and extension of the model by other researchers.

We appreciate that the model is complex. We provide a full README on the model fitting process on our Github (https://github.com/Riadsala/FoMo) as well as example scripts: we now highlight this more clearly on L828.

Introduction

1. Individual differences, development, and MVT-related findings (p. 4, lines 363–366)

On page 4, the authors discuss individual differences in visual foraging and note that FoMo can detect such differences, particularly in developmental contexts (lines 363–366). When discussing Marginal Value Theorem–related aspects, however, the Introduction does not note that evidence for developmental differences in MVT-related measures in humans is currently limited (e.g., Gil-Gómez de Liaño et al., 2022; Gil-Gómez de Liaño & Wolfe, 2022). As a purely optional contextual clarification, the authors could briefly mention that developmental differences are not always observed in MVT-related outcomes. This contrast may be particularly interesting given evidence that spatial organization shows developmental differences (e.g., article 56 cited in the manuscript), and that spatial organization may predict leave/stop decisions in visual foraging better than MVT, as shown in a recent work (Bella et al., 2025). I emphasize that this is intended as a conceptual suggestion rather than a request to include specific citations. If the authors find it useful, acknowledging this contrast either in the

Introduction (p. 4) or later in the Discussion (p. 17, lines 1151–1158) could further strengthen the theoretical motivation for the spatial parameters introduced in the model. (References for these studies can be provided below if the authors find this suggestion useful.)

Thank you for your points here: we have now included references to these studies both in the introduction (L387) and the discussion (L1210).

1. Minor typographical issues in the intro

We have double checked and fixed some minor typographic issues in the introduction.

2. Page 5, line 344: the phrase "differences in" appears to contain an extra "in" and may need correction.

Fixed.

3. Page 7, line 508: the word "next" appears to be unnecessary.

Fixed.

4. Figure 4 caption (page 7), third line: "fleixbly" should be corrected to "flexibly."

Fixed.

Methods

1. Justification of the value of K (p. 8, line 555; p. 9)

Around page 8 (line 555), it is stated that K is determined manually, and later (on page 9) it is specified that K = 20. However, the rationale for choosing this specific value is not explained. Although the Results section later shows that different values of K do not substantially affect the outcomes under the current model, the initial choice of 20 still appears somewhat arbitrary without further justification. I suggest briefly explaining why this value was selected, either at its first mention (p. 8) or when it is specified numerically (p. 9). Providing this explanation would improve transparency and prevent confusion for readers.

We have included a new Figure 5 (page 9) that illustrates the difference in the directional weighting between kappa = 10, 20 and 40, with the following figure caption: *"Illustration of three different \kappa values. We used a value of \kappa=20 in the work presented*

here, which strikes a balance between minimising the overlap between our four components without assigning angles around the oblique directions all to 0."

2. Use of the terms "trial" and "patch"

A more general issue that applies to both the Methods and Results sections concerns the use of the terms trial and patch. My understanding is that these concepts are closely related, and that a trial may effectively correspond to what is typically referred to as a patch in the foraging literature. However, this equivalence is not explicitly stated. Clarifying this relationship at some point in the Methods would be helpful. If the terms are not strictly equivalent, a brief explanation of the distinction would also be beneficial. This issue becomes particularly confusing on page 10, where trials are discussed and the phrase "as opposed to in patches" (line 683) may lead to ambiguity, at least for readers with a background in foraging theory.

Thank you, we have now added a sentence to clarify at the beginning of the methods (L410): in brief, in the studies we look at in this manuscript, a patch is equivalent to a trial. We have reworded "as opposed to in patches" to "as opposed to clumped" which is a better description with regards to the above definition, as the spatial organisation difference still happened within a trial/patch.

Results

1. Clarification of chance-level performance (p. 11, line 801)

On page 11 (line 801), the text refers to performance being "around 50%." While this information is reported in Table 3, I suggest adding a brief clarification such as "when chance performance is below 0.12 in all cases" (or similar wording). This additional context would make the result easier to interpret at first glance. The point is explained clearly later in the Discussion, but including it here would make the Results section more informative and self-contained.

Suggestion implemented.

2. Reminding the reader of model differences across the Results

At some points in the Results section, it becomes slightly difficult to keep track of what each model variant contributes. In particular, Model 1.3 (arguably the best-performing model and the one that incorporates absolute direction) could be briefly reintroduced or reminded to the reader at key points throughout the section. This would help maintain clarity when interpreting the comparative results. This is a very minor suggestion, and the authors should feel free to adopt it only if they find it useful.

Thank you for this point, we have now added reminders throughout the results section to aid clarity.

3. Figure 6 caption

The caption of Figure 6 would benefit from additional detail. Although the main text explains the figure, it would be helpful if the caption itself included more information, particularly regarding what is being measured on the axes in the lower panels. In addition, briefly reminding the reader what parameters such as b_a, b_s, etc., represent in the four upper panels would improve accessibility and allow the figure to stand more independently from the text.

We have updated the axis labels in the top plot to be clearer. We have also extended the caption to give more detail.

4. Table 4 caption

A similar comment applies to the caption of Table 4. While some information is provided, adding a bit more detail to explain what each value represents would facilitate interpretation, especially for readers who may consult the table without reading the surrounding text in detail.

Caption improved as requested.

5. Clarity of Figures 7 and 9

In Figure 7, it would be helpful to explicitly state in the caption that the white lines represent the search path and that the triangles correspond to targets (this is my interpretation). Without this clarification, the intended message of the figure is difficult to grasp. A similar issue arises in Figure 9. These are minor presentation issues, but clarifying these elements would improve the overall comprehensibility of the results.

Both captions have been updated as suggested.

Overall, these are minor figure-related suggestions aimed solely at improving clarity and interpretability; addressing them would further strengthen the presentation of the Results section.

Discussion

1. Search strategies as a potential future direction

The Discussion is very well written and thoughtfully connects the findings to the broader literature. One aspect that I found particularly stimulating is the discussion of search strategies. This prompted the thought that it might be valuable in future work to explicitly manipulate or simulate different search strategies (e.g., spiral search, left-to-right scanning resembling reading) and examine how the model responds to these organizational patterns. This could involve instructing participants to adopt specific strategies or implementing such strategies in simulations. I offer this purely as a conceptual suggestion.

Thank you, we have now referred to the idea of directly manipulating search strategies in the discussion (see L1118).

2. Dynamic foraging environments

In the section on Future Directions, it might be worth briefly mentioning dynamic foraging environments. There is a substantial literature on dynamic in-movement foraging, and a short discussion of how the current framework could be extended to situations in which targets or environments change and move around could broaden the scope of the paper without requiring additional analyses. Again, this is intended as a suggestion rather than a requirement.

Thank you for this suggestion: we agree that considering dynamic and more realistic environments is an important future goal for FoMo (and is also something raised by R1). We have added a paragraph to the end of the discussion highlighting this (L1211), as well as referring to one of our recent pre-prints which is an initial attempt to apply a version of FoMo to a (very simple) more realistic foraging experiment. We look forward to being able to develop these ideas as more secondary datasets become available.

3. Two-dimensional versus three-dimensional environments

Finally, while the focus on two-dimensional space is entirely appropriate given the experimental paradigms considered, it may be useful to briefly acknowledge that real-world foraging often occurs in three-dimensional environments. Highlighting this point would help situate the current model as an important first step, while recognizing potential extensions to more ecologically realistic settings.

See above: discussion of this is now included in the future directions section.

TO CONCLUDE, I reiterate that this is a very strong manuscript that makes a valuable and timely contribution. The comments above are minor and primarily intended to improve clarity and accessibility. In my view, the article clearly merits publication and will be of considerable interest to the scientific community.

Refs.

Bella-Fernández, M., Suero Suñé, M., Ferrer-Mendieta, A., Gil-Gómez de Liaño, B. (2025). One factor to bind them all: visual foraging organization to predict patch leaving behavior with ROC curves. Cognitive Research: Principles & Implications, 10, 16. https://doi.org/10.1186/s41235-025-00624-7

Gil-Gómez de Liaño, B., Muñoz-García, A., Perez-Hernandez, E., & Wolfe, J. M. (2022). Quitting rules in hybrid foraging search: From early childhood to early adulthood. Cognitive Development, 64, 1-14. https://doi.org/10.1016/j.cogdev.2022.101232

Gil-Gómez de Liaño, B. & Wolfe, J. M. (2022). The FORAGEKID Game: Hybrid-Foraging as a new way to study aspects of executive function in development. Cognitive Development, 64, 1-14. https://doi.org/10.1016/j.cogdev.2022.101233

Reviewer #3

This is an interesting paper describing the next version of the authors' FoMo (Foraging Model). Version 1.3 includes a directional component that helps in the prediction of what item gets picked next in a foraging task. The model does a very decent job of predicting behavior in several datasets. Someone other than me should vouch for the computational details of the model but it looks solid to me.

I have a few issues, large and small

1) My main concern is that FoMo is, at present, a big hammer that is hitting a rather small nail. There is a lot of modeling here for purposes of explaining a rather restricted set of data. FoMo "currently assumes exhaustive foraging". This is fine for analyzing Kristjansson-style data where Os are instructed to search exhaustively for two types of target. I suspect that non-exhaustive foraging is much less systematic and, indeed, apparently FoMo does not do as well with the Tagu data which (I gather) is non-exhaustive. So, I worry if FoMo might be a lot of model for showing that Os tend to search very systematically when forced to find everything.

We agree that one of the current limitations of FoMo is that it is largely optimised for exhaustive foraging, although it is a little unclear whether that is the key limitation for the Tagu dataset (there are a number of slightly different things about it, including the value-no value manipulation, and the fact that we have collapsed the number of target

categories - in the ‘low value’ condition there were actually several colours of target possible, but at present, FoMo only works with two target categories - though see below!) In general, we have taken the approach of trying to develop a robust framework that we then extend to be able to tackle more general problems. As mentioned in our response to R1, we are beginning to get work uploaded to pre-print servers e.g. work led by our PhD student (https://osf.io/preprints/psyarxiv/v3jz4_v1) that shows that FoMo is applicable to domains beyond Kristjansson style tasks.

2) At the very least, you should be saying more about the distinction between exhaustive and non-exhaustive search tasks. For instance, in section 2.5, you should be more explicit about the instructions in the tasks that are being modeled I don’t think you mention that the Tagu task is non-exhaustive. That point comes up later.

Thanks, we have now updated Section 2.5 to make it clear that this is a non-exhaustive foraging task (see L711).

3) Switching behavior is also quite different (I think) in non-exhaustive foraging: e.g. Wolfe, J. M., Cain, M. S., & Aizenman, A. M. (2019). Guidance and selection history in hybrid foraging visual search [journal article]. Atten Percept Psychophys, 81(3), 637-653. https://doi.org/10.3758/s13414-018-01649-5

We are actually working on extensions to FoMo that predict patch-leaving behaviour at the moment, and we would be very excited to collaborate with you on being able to access data such as that from your paper above to help test how well FoMo can perform when trying to model these tasks. As discussed above, we are trying to build up the model slowly in order to be able to test it rigorously, and therefore we intend for questions around patch-leaving to be a separate strand of work.

4) Oh, and that paper looks at switching between four target types. How would FOMO do with that?

This is also an area we are actively working on - theoretically, it is a relatively straightforward extension, involving building in transition matrices for different types of switch (A-B, B-C, C-A etc.) Again, we would be delighted to be able to test out how well it works on your data (we can’t currently see an open-source version of the data for the paper above, but please do let us know if we’ve missed something).

5) I wonder how well a really simple nearest neighbor model do with standard Kristjansson data? Such a model would have a switch probability (low for conj, higher for feature) and then just a simple nearest neighbor rule. That might be a nice benchmark to show how FoMo can do.

This is a really great point and is something we have implemented in https://osf.io/preprints/psyarxiv/v3jz4_v1. In brief, a nearest neighbour model does seem to work well in that task (which is even simpler than Kristjansson-style foraging, as there is only one target type) but FoMo can still outperform it when using a slightly altered decision rule (essentially choosing the target with the maximum weighting).

Some smaller points

6) P3 says , about our driller/scanner work; "radiologists who adopted a 'scanning' strategy, searching an entire 2D scan before moving in depth performed more poorly, making more search errors, compared to participants who had a 'drilling' strategy, restricting their eye movements to one portion of the image and scrolling through in depth (37)." I did not go back to check, but my recollection is that you are right in numerical terms but that the difference is not statistically reliable. Hard to get the required power with radiologist observers.

Thanks: we have edited this to say that scanners had a lower true positive rate for detecting nodules compared to drillers (see L196) which the article suggests was statistically supported in your sample.

7) On P7, I could have used a few more words justifying the negative exponential

We have added this on L521-526.

8) And on P8 "Constraining K", I could use some more explanation about the added parameters. What are they doing?

We have tried to increase clarity here, particularly in the previous section where the parameters are described in more detail: in brief, for each direction k, we fit theta and kappa values that give the strength of direction preference and the 'concentration' (or spread) around this direction. The 'constraining kappa' section refers to the fact that trying to fit both theta and kappa simultaneously is challenging, and therefore in practice, we manually set kappa and therefore only the theta parameters are free to vary during the model fit. We have also included more details in Table 1 of the different parameters and Figure 5 has also been edited to show further how we determined the value of the kappa hyperparameter.

9) For the FoMo description, I would add a table of terms as an appendix or something. I can't keep them in memory. (b(a), b(s)....etc)

We have now included Table 1, which is an overview of notation used to define FoMo, which we hope aids model understanding.

10) Fig 5 is very small and hard to read.

We have increased the size of this figure (now Fig 6).

11) P14 says “with most participants beginning in the top right-hand corner” Not top left as in reading? See figs 1 and 7?

Apologies, you’re absolutely right - we meant the top left corner and this has now been corrected.

In sum, the FoMo project is a good and rigorous one. I think the current version is a bit limited in its scope. I hope that FoMo2 predicts quitting times, for example. Nevertheless, FoMo represents one of the best efforts to formally model human foraging behavior.

Thanks

Jeremy Wolfe (obviously signed review)
