# Supplementary material for: FoMo: A unifying theory of visual foraging
Source: PLoS Comput Biol. 2026 May 12;22(5):e1014266. doi: 10.1371/journal.pcbi.1014266 (PMC13186390; doi:10.1371/journal.pcbi.1014266)
Supplement: Attachment — Submitted filename: response_to_reviewers_2.pdf [file pcbi.1014266.s004.pdf]

We thank the reviewers for their very helpful comments on the manuscript. Please see our responses below (in red). Page/line numbers refer to the track changes version of the manuscript (where the additions/edits we have made appear in blue).

Reviewer #1: I have no further comments and recommend publication

Reviewer #2: Thanks for all changes, I think the paper is ready to be published.

Reviewer #3: This is a fairly lightly revised paper describing the latest version of the detailed FOMO model of human visual foraging. I still wish that the model had been deployed on a richer set of phenomena. The discussion in the Response to Review is quite interesting, pointing in more wide-ranging directions than are found in the revisions to the paper. Nevertheless, the existing paper will be a useful documentation of the current state of an evolving model.

A few minor bits

P6 says These four features are illustrated in Figure 3. These are more formally defined in Section 2.1, ..." Are those the right section #s? I think you mean maybe 3.2.

Apologies, the section numbering got out of order because of needing to add an author summary. We have now removed the number from the author summary section, which should mean everything is now correctly labelled again.

P 11 says "This dataset is inexhaustive; participants had to collect a certain number of points on each trial, but this did not necessarily correspond to collecting all targets on the screen."

This makes it sound like the dataset is incomplete. Maybe something like "The Tagu et al task did not require that Os collect all targets in one patch before moving on to the next patch. They were required to collect (at least??) N points per patch"

We have now reworded to "The task did not require that subjects collect all targets in one trial before moving onto the next trial: instead, they were required to select enough targets to reach a pre-specified number of points. Trials automatically ended once they had reached the required number of points." (L713).

Now that I can see Fig 6, I don't fully understand it. The numbers on the axes on the lefthand figure are accuracy? The secondary Y-axis is a reference to a dataset? And what is HPDI? (not High-Pressure Direct Injection, I am guessing)

Yes, you are correct re. the axes - we have updated the axis labelling of this figure to make these points clearer. We have also spelled out the acronym (Higher Posterior Density Interval) - essentially, this is the Bayesian equivalent to a confidence interval, displaying the range containing a particular percentage of probable values (i.e. in this case, the portion of the posterior density that contains 95% of the values).
